# Incidental neuroendocrine tumor of a complete subserosal appendix: an unusual presentation of a rare anatomical variation. A case report and review of literature

**DOI:** 10.1186/s12893-021-01429-3

**Published:** 2021-12-16

**Authors:** Stalin Isaías Cañizares Quisiguiña, Lucía Vanessa Guamán Maldonado, Iván Marcelo Hidalgo Jaramillo, Tatiana Paola Borja Herrera, Cecilia de los Ángeles Carrión Guzmán

**Affiliations:** 1 Escuela de Medicina, Colegio de Ciencias de La Salud COCSA, Universidad San Francisco de Quito USFQ, Quito, Ecuador; 2 Hospital de los Valles, Quito, Ecuador; 3 Instituto de Investigaciones en Biomedicina, Universidad San Francisco de Quito, Quito, Ecuador; 4 Servicio de Pediatría, Hospital Metropolitano, Quito, Ecuador; 5 Servicio de Patología, Hospital Solca de Quito, Quito, Ecuador; 6 Servicio de Patología, Hospital Vozandes, Quito, Ecuador

**Keywords:** Complete subserosal appendix, Intramural appendix, Intracecal appendix, Neuroendocrine tumor, Acute appendicitis

## Abstract

**Background:**

Appendix’ anatomical variations are a rare occurrence which can mislead diagnosis and delay appropriate treatment.

**Case presentation:**

We present a 9-year-old female patient that came with a clinical picture compatible with acute appendicitis. However, a cecal mass was identified instead of an inflamed appendix during surgery. Therapeutic decisions were extremely challenging due to clinical deterioration and an uncertain etiology. Only the histopathology report revealed the presence of a complete subserosal appendix which was responsible for the entire symptomatology. Here, we review all case reports regarding intramural, intracecal or subserosal appendixes. A discussion of the general approach to this specific case and the importance of consensual diagnostic criteria for these specimens are also presented. At last, an incidental finding is exposed and final treatment options are discussed given the overall presentation.

**Conclusions:**

Considering these variants would guide physicians towards a more accurate approach to similar clinical pictures and hence an improved long-term prognosis.

## Background

Acute appendicitis is one of the most common surgical emergencies worldwide [1]. It is an acute inflammatory process of the mucosal layer of the appendix that expands toward the serosa and is most frequently caused by luminal obstruction due to either lymphoid hyperplasia in children or a fecalith in adults [2–4]. Other causes include fibrous obliteration, eosinophilic infiltration, parasites, actinomycosis, tuberculosis, Crohn’s disease, endometriosis, diverticulitis, foreign body, and benign or malignant tumors [2, 3, 5–8].

Among gastrointestinal neoplasms, appendiceal tumors are rare, presenting in only 1 percent of appendectomies’ specimens [2, 3, 5, 9]. The vermiform appendix is the second most involved organ by neuroendocrine tumors [NETs] in the gastrointestinal system [10, 11]. These are detected in up to 2.27% of patients diagnosed with acute appendicitis and in up to 2.3% of patients undergoing incidental appendectomies [8, 11–14]. The World Health Organization classified NETs into well differentiated (low, intermediate, and high-grade neuroendocrine tumor) and poorly differentiated tumors (high grade neuroendocrine carcinoma) based on ki67 proliferation index and number of mitosis [11]. In general, 95% of appendix NETs are smaller than 2 cm and show a low (4%) metastasis potential [11]. As a result, most of them are resolved with simple appendectomy and no adjunctive surgical procedure or long-term follow up is required [11, 14].

The vermiform appendix is usually found on the right inferior abdominal quadrant, emerging from the posteromedial region of the cecum, and settling inferior to the ileocecal union. However, its location varies widely among surgical specimens. The orientation of the tip of the appendix defines its anatomic position [15, 16] either medially, caudally, laterally or posterior regarding the cecum’s blind end [17]. Retrocecal appendixes direct upward behind the cecum and are in fact the most common variant [15, 16]. Alternatives include subcecal (24.4%), post-ileal (14.3%), pelvic (9.3%), paracecal (5.8%), pre-ileal (2.4%); and some other less common presentations (0.27%) [16] such as subhepatic, intrahernial, left-sided and mesoceliac appendixes [17].

Out of these uncommon anatomic variations, intracecal and intramural appendix are even rarer. To the best of our knowledge, there are only three cases reported worldwide [1, 18, 19]. The first intramural appendix was reported in 1972 without any profound anatomic or histologic description [19]. In 1983, an intracecal appendix was diagnosed after ruling out appendix agenesia, intussusception and the possibility of an intramural presentation [18]. It was described as a “peeled seedless grape” due to the absence of a serous layer. The authors pointed out that an intramural variant required of an appendix localized within the cecal wall, internally covered by the cecum serosa and externally coated by the peritoneum [18]. In 2018, Chauhan and Anand complemented these criteria and proposed an exhaustive definition for an intracecal appendix: (1) the base of the appendix should not be distinguishable from the cecum; (2) if present, local inflammation should not fully explain the adhesion between the appendix and the cecum; (3) there is no specific mesoappendix; (4) the vascular supply tends to adapt to the anatomic variation; and (5) the cecum tissue clearly encloses the appendix [1]. It seems that the characterization for intracecal appendixes by Abramson et al. was misleading and the one from Chauhan and Anand might not fully contain the whole spectrum of intracecal appendixes. Under these circumstances, a joint analysis of current reported cases would be beneficial for the consolidation of gross indicators that help identify these clinical pictures during surgical procedures and define precise histopathological diagnostic criteria.

## Case presentation

A 9-year-old female patient arrived at the emergency room because of a 36-h history of intermittent right lower abdominal pain, anorexia, vomit, and quantified high-grade fever. She had no pathological personal or family history of interest. On examination, the right iliac fossa was tender to palpation and no frank peritoneal signs were observed. Initial laboratory evaluation showed leukocytosis, neutrophilia, and an elevated C-reactive protein. Ultrasonography of the abdomen was inconclusive. A heterogeneous lesion of 40 × 37 mm within the colon, no appendix and some swollen mesenteric nodes of at least 10 mm were reported. A complementary abdominal CT scan revealed findings suggestive of ileocolic intussusception with an invagination area of approximately 6.6 × 4.9 cm. After surgical consult, the patient underwent an exploratory laparoscopy that required laparotomy conversion. A well-defined, 5 cm mass at ileo cecal valve and multiple hard pericecal lymph nodes were observed. Preserved permeability between the ileum and colon, complete integrity of the cecum wall and lack of vermiform appendix were also reported. The possibility of an auto-digested appendix and a cecal tumor were discussed. At this time, surgeons decided to resect retrocecal and pericecal lymph nodes and send these samples to pathology before any further intervention. The patient was admitted to the inpatient floor where antibiotic therapy based on ampicillin sulbactam, and metronidazole was initiated. The oncologist department was consulted and complementary laboratory exams including liver and renal function tests, uric acid, electrolytes, lactic dehydrogenase, and quantiferon-TB tests were ordered. Only lactic dehydrogenase was altered. A chest x-ray ruled out mediastinal masses. No alarming findings were reported. However, the patient presented gastric distension, abdominal pain and fever by the second hospitalization day. The content inside the suprapubic JP drain changed from a serohematic aspect to a dense cloudy fluid. A culture and cytochemical analysis of peritoneal fluid was performed without significant results. CBC showed mild leukocytosis and neutrophilia. Reactive C-protein remained elevated. Two blood cultures and an urinalysis were negative. Due to the uncertainty of the etiology of her clinical picture, infectology decided to change antibiotic therapy to piperacillin/tazobactam and amikacin. An abdominal x-ray showed air fluid levels in the small bowel and a colonic distention projected at mesogastrium. Gastroenterology suggested initiating bowel rest and placing a central line for parenteral nutrition.

After five more days, elevated inflammatory markers, abdominal distension and pain, and the unusual JP drain aspect persisted. A new ultrasound confirmed that the mass and surrounding area had the same aspect as days before. The histopathological description of paracecal-retrocecal lymph nodes and the sample of mesenteric omentum obtained during the first intervention failed to detect neoplastic cells. Macroscopically, three encapsulated lymph nodes from 0.8 to 2 cm were received. Their physiological architecture was preserved; secondary lymphoid follicles with hyperplastic germinal centers containing macrophages with cellular debris were reported. The interfollicular population was polymorphic and contained frequent large cells with prominent immunoblast-like nucleoli. Other areas showed sinusoidal histiocytosis with eosinophils and neutrophils. There was fibrosis with a predominantly neutrophilic mixed inflammatory infiltrate that spread to neighboring adipose tissue in the periphery of the nodes. The immunohistochemical study confirmed the presence of follicular dendritic cells and B lymphocytes in the germinal centers (CD23 + + +/+ + + and CD20 + + +/+ + + respectively), T lymphocytes in the mantle zone (CD3 + + +/+ ++), macrophages in germinal centers and sinusoidal area (CD68 +/+ ++). Frequent CD30 + + +/+ + + immunoblasts and actin + + +/+ + + myofibroblasts within areas of fibrosis were also observed. EBV study using EBER in situ hybridization was negative. Ziehl Neelsen and PAS did not show any pathogen. The 22 × 0.6 cm omentum sample showed fibrous thickening of the septa and the presence of a mainly lymphocytic infiltrate. Fibrino-leukocytic material was also seen in the serosa. Pathologists concluded the possibility of an unspecified acute versus chronic epiploitis, lymphadenitis and serositis. Nevertheless, due to her unfavorable clinical evolution and the elevated inflammatory markers, a second surgical intervention was decided. The patient underwent an omentectomy and resection of approximately 40 cm of terminal ileum, cecum and ascending colon. Pericolonic lymph nodes were resected as well. A sample of a collection observed near the cecum was taken for culture and cytochemical studies before aspiration and drainage. After surgery, the patient remained hemodynamically stable, without abdominal pain or distention. A nasogastric tube was placed and parenteral nutrition continued. The peritoneal fluid analysis was negative. Improvement in inflammatory markers lead to amikacin discontinuation. And by the fifth postoperative day, JP drain, and nasogastric tube were removed. Later, a regular diet was successfully initiated, and the patient was finally discharged.

**Fig. 1:**
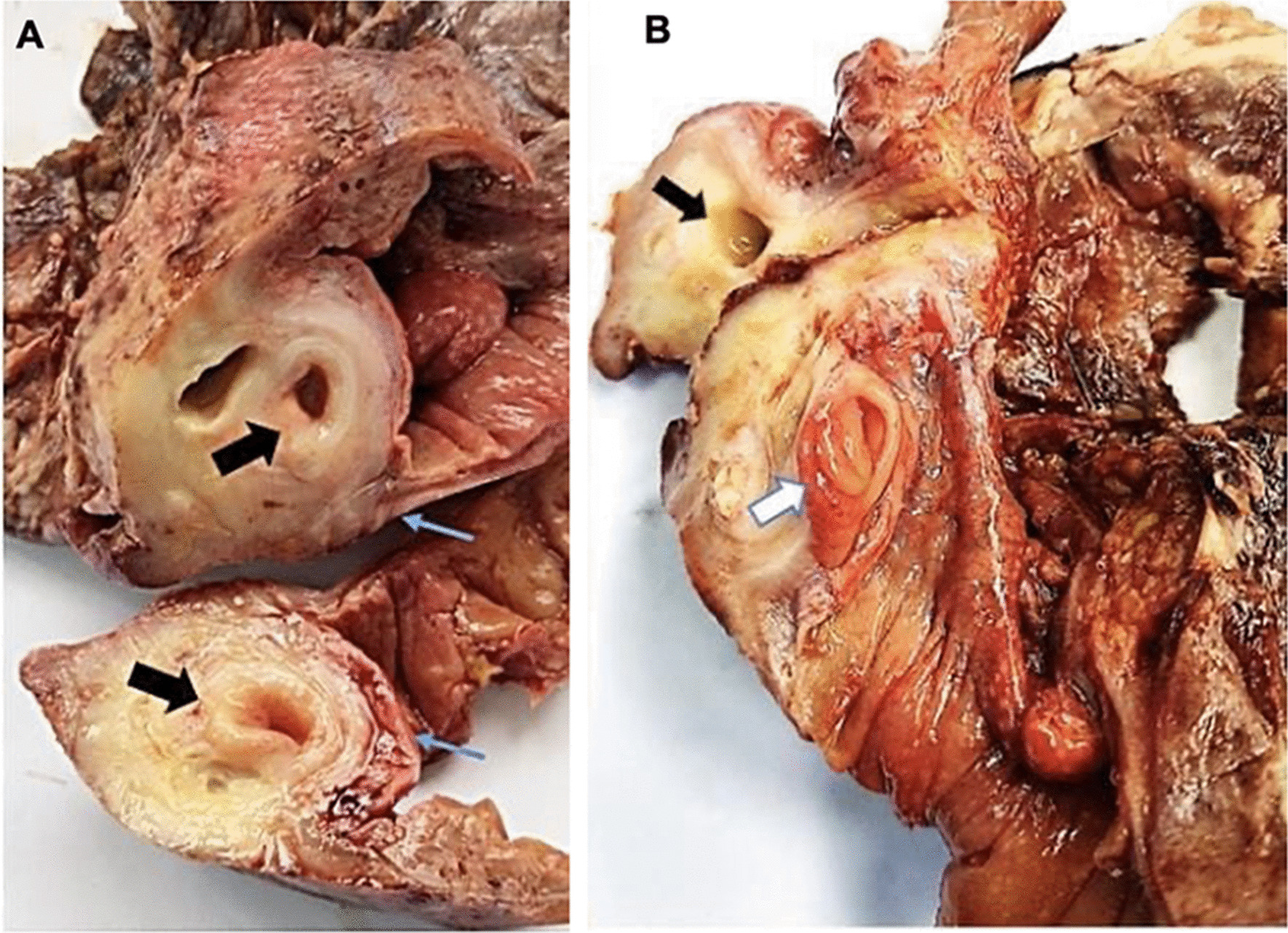
Macroscopic transverse cross-section view of the cecum sample. **A** shows a subserous appendicular lumen *[thick black arrow].* The appendix is completely surrounded by the serous layer *[thin blue arrow].* **B** shows the appendicular base *[thick white arrow]* and a cavitated area within the cecal wall, filled with purulent material, probably due to the local inflammatory process *[thick black arrow]*

**Fig. 2:**
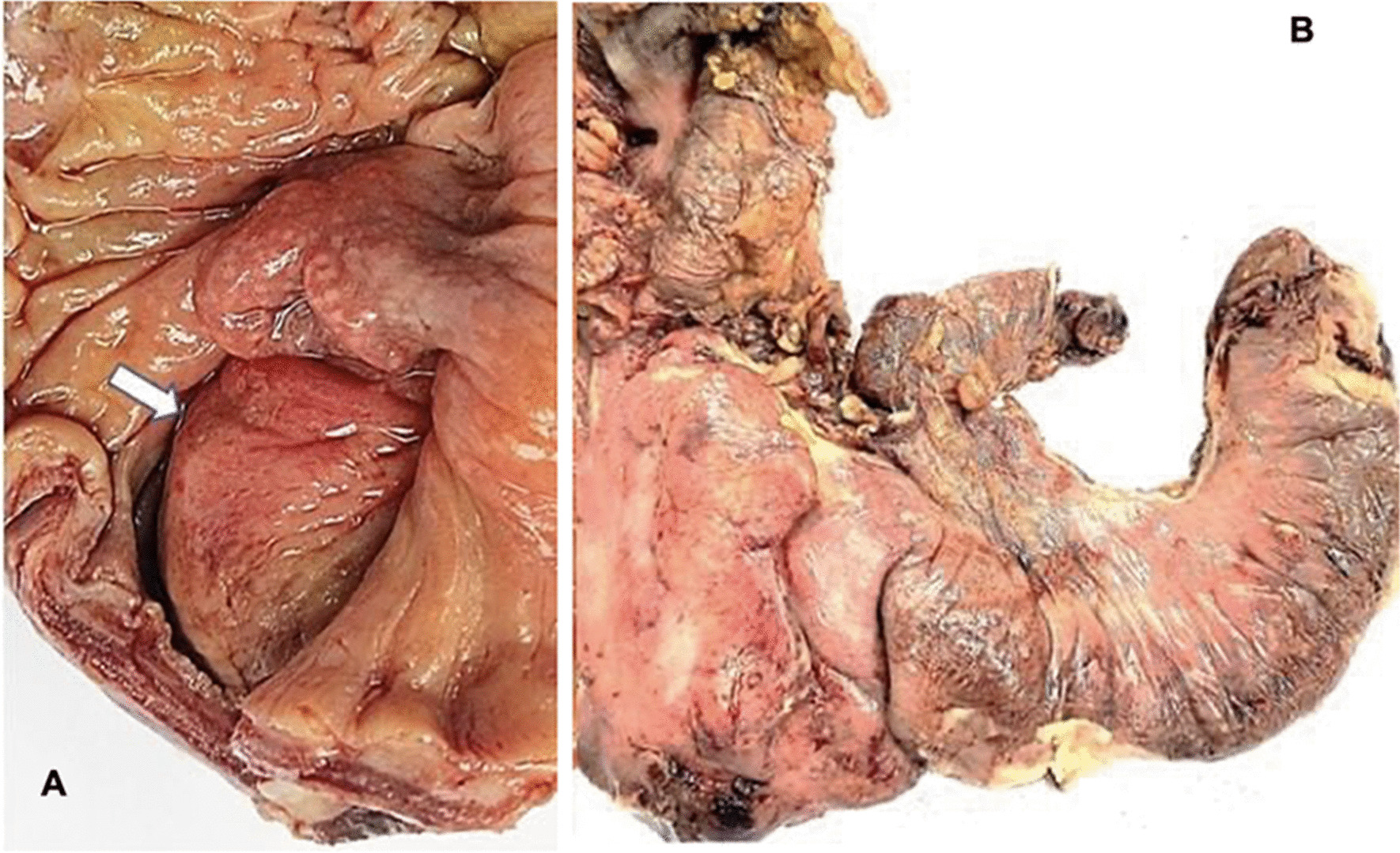
Right colon sample. **A** shows a raised cecal mucosa that gives the impression of a cecal mass. **B** shows purulent fibrinous gleas on its surface. No cecal appendix is evident in either projection

**Fig. 3:**
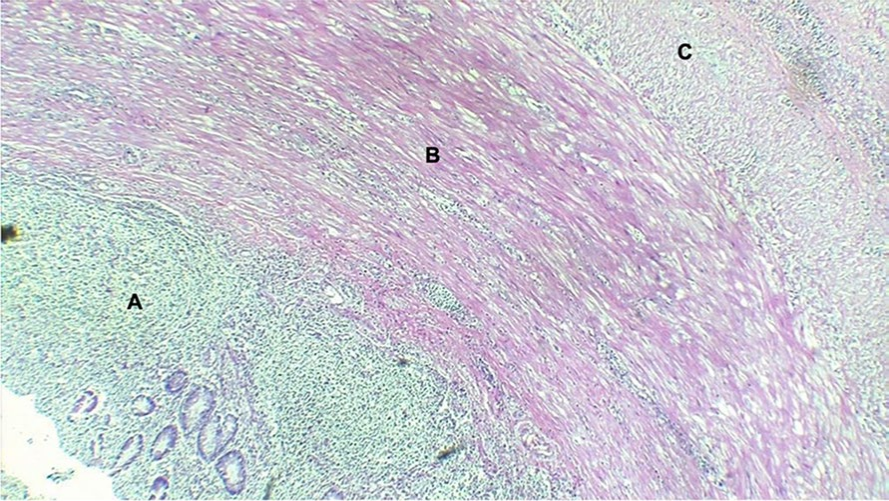
H&E. Histological transverse section of the completely subserous vermiform appendix. This sample does not share any layer with the cecum wall. Both images expose the integrity of its independent mucosa (**A**), muscularis propria (**B**) and serosa (**C**)

**Fig. 4:**
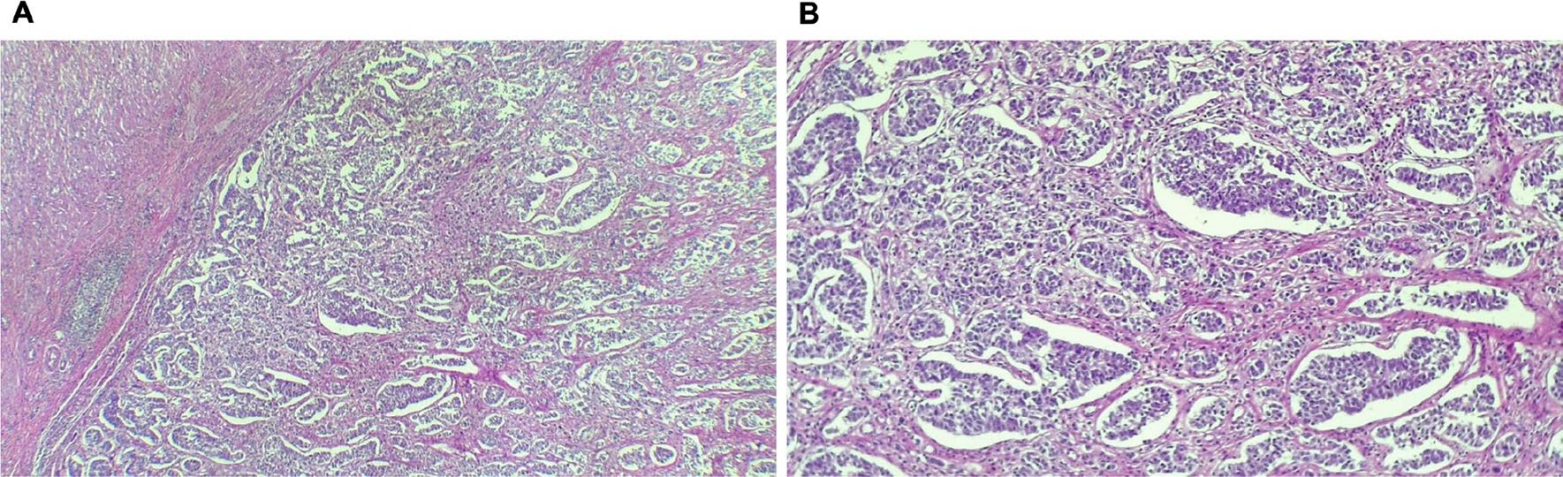
H&E. Vermiform appendix. (**A** [4×]) exhibits organoid and trabeculated cell aggregates similar to neuroendocrine patterns. (**B** [10×]) verifies the organoid and pseudoglandular nature of these cell nests. They infiltrate the appendix’ muscularis propria

**Fig. 5:**
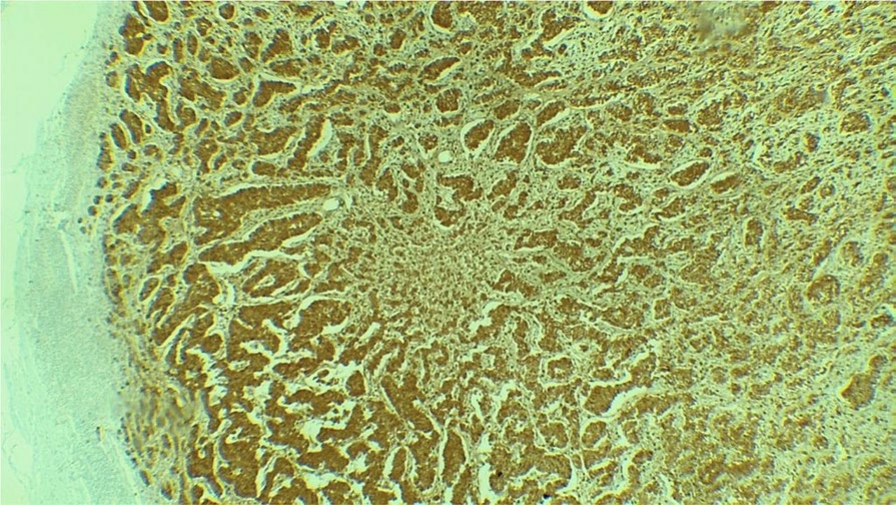
IHQ. Chromogranin A. Intense cytoplasmic positivity of tumor cells that confirm the neuroendocrine lineage

The histopathological final report described an 8 cm ileal segment, and a 14 cm ascending colon including the cecum with a diameter that ranged from 1 to 3 cm. The external surface was covered by a pinkish-gray serosa with fibrinopurulent material over the ileocecal area. A completely subserous dilated appendix was identified within the cecum wall. It contained a white-yellowish purulent material at the tip (Fig. 1). A well-defined nodular lesion of approximately 1.5 cm was also identified (Fig. 2). The mucosa of the cecum was pink while ileal mucosa had a granular appearance. Nine nodules, which measured between 0.3 and 3 cm, were isolated from the surrounding area. The 12 × 4.5 cm omentum sample had no palpable nodes. A second omentum sample showed multiple whitish irregular fragments of bland tissue that measured between 0.8 and 1.5 cm. Microscopically, the histological findings of the fourteen isolated lymph nodes were compatible with follicular hyperplasia. The subserosal cecal appendix showed transmural necrosis and perforation causing leakage of purulent material and an acute inflammatory reaction of the surrounding adipose tissue which extended up to the cecal and ileal serosa. All layers of the appendix were independent and unrelated to the cecum wall (Fig. 3). The distal portion of the appendix showed the proliferation of cellular nests that were composed of round uniform nuclei with a “salt and pepper” appearance (Fig. 4). No mitotic activity was evidenced. It seemed to infiltrate the muscular layer of the appendix and reach a diameter of 1.5 cm. No lymphovascular or perineural invasion was observed. Disease free margins were reported. Ileum dissection showed Peyer’s patches hyperplasia with wide germinal centers. Tumoral cells’ immunochemical studies showed a Ki67 proliferative index of 2%, a positive (+ + +/+ + +) cytoplasmic granular pan-cytokeratin, a positive (+ + +/+ + +) cytoplasmic chromogranin and a negative synaptophysin reaction (Fig. 5). Pathologists concluded the presence of an incidental well differentiated neuroendocrine tumor grade I pT1 pN0 at the tip of the appendix in the middle of a clinical picture caused by an acute necrotizing appendicitis of a complete subserosal appendix. Due to the stage, no further intervention was required. She fully recovered in subsequent controls. Nevertheless, correct management of short bowel syndrome will become a key feature for the preservation of her future quality of life.

## Discussion

During our patient’s first surgical procedure, physicians found a 5 cm mass at the ileo-cecal valve instead of an inflamed appendix which was primarily expected by the clinical picture. The presence of the adjacent congested lymph nodes raised the suspicion of a neoplastic process. And given the lack of visible appendix, the probability of an inflamed appendix or any related complication to acute appendicitis were dismissed. The likelihood of the mass being responsible for her current symptomatology was superior. During the second intervention, they decided to resect the portion of the involved bowel because of her notorious clinical deterioration. After the procedure, the patient significantly improved giving the impression that the complicated neoplastic mass was a plausible explanation for her clinical picture. Nevertheless, the histopathological results revealed that, in fact, the mass in the cecum wall corresponded to a completely subserous inflamed appendix which was the origin of the patient’s worsening. Given the circumstances, taking into consideration the possibility of a neoplastic process and assuming the absence of the vermiform appendix was a reasonable approach. Appendix agenesia constitutes a rare anatomic variation that has been reported in 1 for every 100,000 laparotomies [20]. It is a diagnosis of exclusion that requires the absence of previous abdominal surgeries [21], the legal confirmation of no preceding appendectomies [22], the lack of an identifiable appendicular base by tracing the three taenia coli on the large bowel wall [16, 22] and the exclusion of the possibility of an intussuscepted, intracecal or intramural appendix [22].

Here, we present a rare congenital anatomic variation that does not entirely meet the criteria exposed above: a complete subserosal appendix. The lack of a depression or pit in the cecum wall, which usually provides evidence of the original anatomic position, ruled out intussusception [18]. The lack of an intraluminal position and the presence of the cecum serosa covering all its trajectory ruled out the possibility of an intramural variant according to Abramson et al. The lack of cecum tissue completely surrounding the appendix ruled out the possibility of an intracecal appendix according to Chauhan et al. [1, 18]. Furthermore, this anatomic variation is more likely not secondary to the local inflammatory process. The physiopathology behind acute appendicitis is complex. The obstruction of its lumen increases intraluminal pressure compromising its irrigation and causing edema of the wall with subsequent invasion by intraluminal bacteria. Depending on the time and extension of the inflammation, acute appendicitis is classified in acute intraluminal inflammation, catarrhal inflammation, acute mucosal and submucosal inflammation, phlegmonous appendicitis and acute necrotizing appendicitis [23]. Among these clinical pictures, a perforated appendix wall induces the creation of an appendicular plastron as a defense mechanism to the local barrier transgression [24]. This is an inflammatory mass created by the adhesion of the appendix to the small intestine, the cecum and/or the epiplon [24, 25]. Our surgical sample showed a fully intact cecum wall with a protruding mass that later was identified as a completely subserosal appendix. Histological analysis later revealed that the appendix did not share any layer with the cecum wall suggesting a congenital anatomic variation. However, because of limited data and the heterogeneity among the scarce case reports in the literature, the histopathological characterization of these specimens to achieve an accurate diagnosis is extremely challenging. Under these circumstances, we propose the following diagnostic criteria for a completely subserosal appendix: (1) the lateral aspect of the vermiform appendix should be fully covered by the cecum serosa; (2) the layers of the appendicular wall must be complete and should not correspond to a prolongation of the cecum wall’s; (3) the base of the appendix is different from the cecum’s; (4) no mesoappendix should be present; (5) macroscopic and microscopic characterization of the appendix and cecum as unrelated and independent organs; (6) intussusception, intramural and intracecal appendix must be excluded. Finally, it is important to clarify that there exists the possibility that some cases reported as appendix agenesia were in fact completely subserosal appendixes that did not undergo histological analysis and therefore remained undiagnosed.

At last, although the mass located at the ileocecal valve actually corresponded to an inflamed subserosal vermiform appendix, an asymptomatic neuroendocrine tumor was incidentally found during microscopic analysis. An analysis of 170 incidental appendectomies performed during living donor hepatectomies between 2005 and 2018 revealed normal histological characteristics in 80.6%, acute appendicitis in 2.9%, low-grade appendiceal mucinous neoplasm in 1.2%, and grade I neuroendocrine tumor in 0.6% of them [4]. Akbulut et al. [4] reported an analysis of sixteen articles regarding the histopathological features of incidental appendectomy specimens which revealed a normal appearance from 22.6 to 89.2%, acute appendicitis in up to 9.2% and various tumors samples in up to 4.2% [12, 13, 26–39]. Based on the cell type, primary appendiceal tumors are classified into epithelial tumor, mesenchymal tumors and lymphomas [14]. Appendiceal epithelial tumors include mucinous, non-mucinous, neuroendocrine and mixed glandular-endocrine tumors [14, 40].

Neuroendocrine neoplasms [NETs] correspond to up to 75 percent of appendix neoplasm [41]. These are most commonly asymptomatic, diagnosed in female patients in their 40 s and found incidentally at the distal portion of the appendix during emergency appendectomies [42–46]. Our 9-year-old female patient was diagnosed with an incidental well differentiated neuroendocrine tumor grade I pT1 pN0 that extended 1.5 cm into the wall. Small tumors, defined as < 2 cm like this one, are more common, unlikely to have metastasized, and have shown a better prognosis [47]. For these patients, overall survival for this group is 100% [48, 49]. The North American Neuroendocrine Tumor Society (NANETS) and the European Neuroendocrine Tumor Society (ENETS) suggest right hemicolectomy plus resection of lymph nodes and ileocolic and right colic arteries only for samples > 2 cm or from 1.0 to 1.9 cm if any of the following is present: deep mesoappendiceal invasion (> 3 mm), vascular invasion, positive or unclear margins, a high proliferative rate (grade 2) or a mixed histology (globet cell carcinoid, adenocarcinoid) [11, 14, 50–52].

A review of the SEER and NCI database reported controversial results regarding the presence of lymph node metastasis and small tumors. It seems that there is a higher-than-expected probability of nodal involvement in tumors from 1.1 to 2 cm, raising the question of whether right hemicolectomy would be appropriate for this group [53]. Despite our patient did not have any of these findings, partial bowel resection was performed due to her impending clinical deterioration and the uncertainty of the diagnosis given the assumption of appendix agenesia. Akbulut et al. [11] proposed that the incidental finding of a tumoral lesion in the gastrointestinal system during a living donor hepatectomy requires a thorough gross examination of its features, its partial or complete resection and an intra-operative frozen examination which will determine the best surgical approach at the moment. In retrospect, considering the possibility of this anatomic variant could have allowed a distinct and more straightforward initial surgical approach.

## Conclusion

The cecal appendix tends to present at a fairly constant location among patients. However, anatomical variations exist. And given the lack of enough evidence to accurately identify these presentations, unnecessary invasive interventions may occur. The correct management of cecal masses under hemodynamic instability is extremely challenging. Considering these variants would guide physicians towards a more accurate approach to similar clinical pictures and hence an improved long-term prognosis. Furthermore, consensus regarding intramural, intracecal and subserosal appendixes is required in order to correctly characterize these anatomic variations that seem to have common backgrounds and slightly differ in small aspects.

## Data Availability

The datasets used and/or analyzed during the current study are available from the corresponding author on reasonable request.
